# [(2*R*,3*S*)-Butane-1,2,3,4-tetraol-κ^3^ *O* ^1^,*O* ^2^,*O* ^3^](ethanol-κ*O*)tris­(nitrato-κ^2^ *O*,*O*′)samarium(III)

**DOI:** 10.1107/S1600536813003255

**Published:** 2013-03-02

**Authors:** Jun-Hui Xue, Xiao-Hui Hua, Li-Min Yang, Yi-Zhuang Xu, Jin-Guang Wu

**Affiliations:** a Chemical Engineering College, Inner Mongolia University of Technology, People’s Republic of China; b Beijing National Laboratory for Molecular Sciences, The State Key Laboratory of Rare Earth Materials Chemistry and Applications, College of Chemistry and Molecular Engineering, Peking University, Beijing, People’s Republic of China; c State Key Laboratory of Nuclear Physics and Technology, Institute of Heavy Ion Physics, School of Physics, Peking University, Beijing, People’s Republic of China

## Abstract

The title Sm^III^–erythritol complex, [Sm(NO_3_)_3_(C_2_H_6_O)(C_4_H_10_O_4_)], is isotypic with its Nd, Eu, Y, Gd, Tb and Ho analogues. The Sm^III^ cation exhibits a coordination number of ten and is chelated by a tridentate erythritol ligand and three bidentate nitrate anions. It is additionally coordinated by an O atom of an ethanol mol­ecule, completing an irregular coordination sphere. The Sm—O bond lengths range from 2.416 (2) to 2.611 (2) Å. In the crystal, extensive O—H⋯O hydrogen bonding involving all hy­droxy groups and some of the nitrate O atoms links the mol­ecules into a three-dimensional network.

## Related literature

For background to the coordination behaviour of sugars to metal cations, see: Gottschaldt & Schubert (2009 ▶). For the crystal structure of free erythritol, see: Bekoe & Powell (1959 ▶). For isotypic structures of the title compound, see: Yang *et al.* (2003 ▶, 2004 ▶, 2012 ▶); Hua *et al.* (2013 ▶).
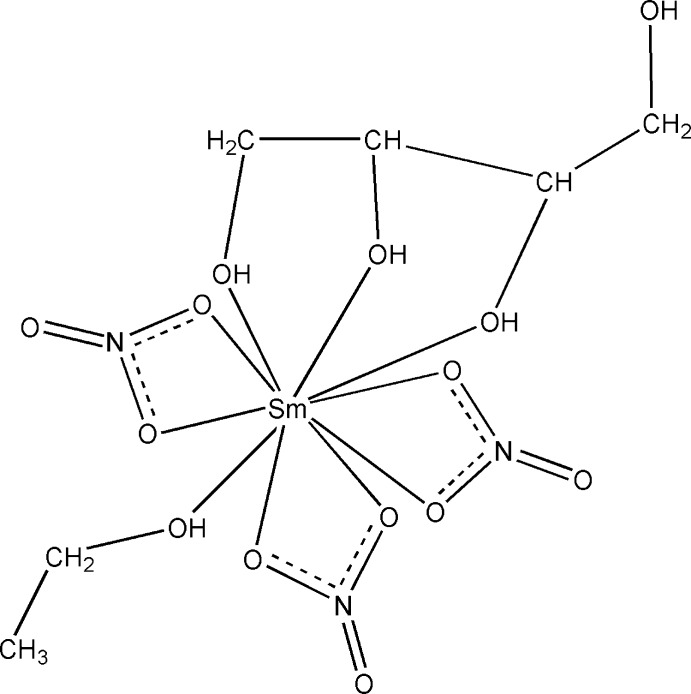


## Experimental

### 

#### Crystal data

[Sm(NO_3_)_3_(C_2_H_6_O)(C_4_H_10_O_4_)]
*M* *_r_* = 504.57
Monoclinic, 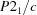

*a* = 7.8537 (16) Å
*b* = 12.875 (3) Å
*c* = 15.252 (3) Å
β = 100.92 (3)°
*V* = 1514.4 (5) Å^3^
*Z* = 4
Mo *K*α radiation
μ = 3.96 mm^−1^
*T* = 173 K
0.27 × 0.21 × 0.16 mm

#### Data collection

Rigaku Saturn724+ CCD diffractometer
Absorption correction: multi-scan (*CrystalClear*; Rigaku, 2007 ▶) *T*_min_ = 0.488, *T*_max_ = 1.000
10349 measured reflections
3446 independent reflections
3315 reflections with *I* > 2σ(*I*)
*R*_int_ = 0.035

#### Refinement

*R*[*F* ^2^ > 2σ(*F* ^2^)] = 0.029
*wR*(*F* ^2^) = 0.057
*S* = 1.22
3446 reflections
218 parameters
Δρ_max_ = 1.33 e Å^−3^
Δρ_min_ = −0.72 e Å^−3^

### 

Data collection: *CrystalClear* (Rigaku, 2007 ▶); cell refinement: *CrystalClear*; data reduction: *CrystalClear*; program(s) used to solve structure: *SHELXTL* (Sheldrick, 2008 ▶); program(s) used to refine structure: *SHELXTL*; molecular graphics: *XP* in *SHELXTL*; software used to prepare material for publication: *SHELXTL*.

## Supplementary Material

Crystal structure: contains datablock(s) global, I. DOI: 10.1107/S1600536813003255/wm2711sup1.cif

Structure factors: contains datablock(s) I. DOI: 10.1107/S1600536813003255/wm2711Isup2.hkl

Supplementary material file. DOI: 10.1107/S1600536813003255/wm2711Isup3.cdx

Additional supplementary materials:  crystallographic information; 3D view; checkCIF report

## Figures and Tables

**Table 1:** Hydrogen-bond geometry (Å, °)

*D*—H⋯*A*	*D*—H	H⋯*A*	*D*⋯*A*	*D*—H⋯*A*
O1—H1⋯O4^i^	0.84	1.83	2.668 (3)	175
O2—H2⋯O7^ii^	0.84	1.96	2.802 (3)	174
O2—H2⋯O8^ii^	0.84	2.54	3.146 (4)	130
O3—H3⋯O12^iii^	0.84	2.07	2.903 (3)	174
O4—H4⋯O8^iv^	0.84	2.09	2.910 (4)	165
O4—H4⋯O6^iv^	0.84	2.55	3.235 (3)	140
O5—H5⋯O11^v^	0.84	2.00	2.827 (4)	167

Symmetry codes: (i) 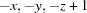; (ii) 
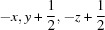
; (iii) 
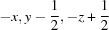
; (iv) 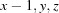; (v) 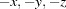.

**Fig. 1.**
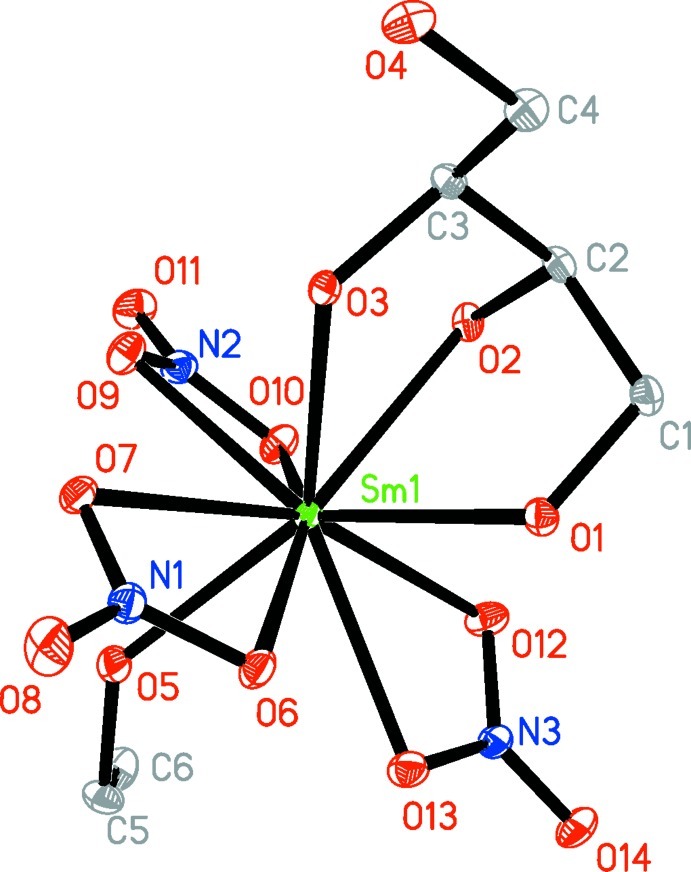
The molecular structure of the title complex with displacement ellipsoids drawn at the 30% probability level. Hydrogen atoms have been omitted for clarity.

**Fig. 2.**
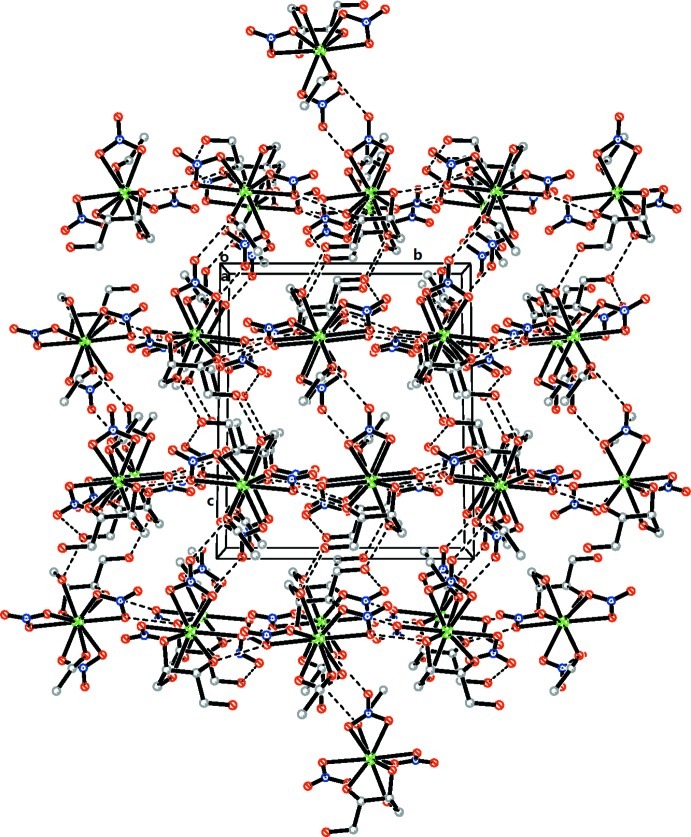
The packing of the title complex, showing hydrogen bond interactions as dashed lines.

## supplementary crystallographic information

### Comment

Metal ions play important roles in the catalysis of numerous chemical and biological reactions. Interactions between carbohydrates (sugars) and metal ions may be involved in many biochemical processes (Gottschaldt & Schubert, 2009). Here the sugar alcohol erythritol was chosen as a model compound to study the coordination behavior of hydroxyl groups to *f*-block metal ions.

The molecular structure of the title complex, [Sm(C_4_H_10_O_4_)(C_2_H_5_OH)(NO_3_)_3_], denoted as SmEN, where E stands for erythritol, N stands for nitrate, is shown in Fig. 1. In the title compound the Sm^III^ cation is 10-fold coordinated by three hydroxyl groups (O1, O2 and O3) from one erythritol molecule, by one hydroxyl group from ethanol (O5), and by three bidentate nitrate ions through O6, O7; O9, O10; O12, O13. The structure of SmEN is isotypic with its Nd, Eu, Y, Gd, Tb (Yang *et al.*, 2003, 2004, 2012) and Ho (Hua *et al.*, 2013) analogues. The Sm—O distances range from 2.416 (2) to 2.611 (2) Å, the average Sm—O distance being 2.499 Å. The C—C—C and the O—C–C bond angles of the central backbone in the free centrosymmetric erythritol molecule are 113° and 107°, respectively (Bekoe & Powell, 1959). After coordination, the C—C—C bond angles are 112.6 (3) and 116.7 (3)° and the O—C—C bond angles range from 104.0 (3) to 111.6 (3)° in SmEN, which indicates a subtle change of the conformation of erythritol.

The extensive hydrogen bond network in SmEN is formed by O—H···O hydrogen bonds from coordinating and uncoordinating hydroxyl groups of erythritol and ethanol and the nitrate O atoms. The coordinating hydroxyl groups O1 of erythritol forms a hydrogen bond with the uncoordinating O4 hydroxyl group of a neighbouring erythritol molecule. The coordinating O2 hydroxyl group forms a bifurcated hydrogen bonds with two oxygen atoms from a nitrate ion (O7, O8). The coordinating hydroxyl group O3 forms a hydrogen bond with an oxygen atom from another nitrate ion (O12). The non-coordinating hydroxyl group O4 is a donor of a bifurcated hydrogen bond to O8 and O6 from one nitrate ion. The ethanol hydroxy group (O5) forms a hydrogen bond with an oxygen atom from a nitrate ion (O11). Details of the hydrogen bonding are given in Table 1 and Fig. 2.

### Experimental

Sm(NO_3_)_3_^.^6H_2_O (3 mmol) and erythritol (3 mmol) were dissolved in 6 ml water and 6 ml ethanol. The solution was put on a water bath, and the temperature was raised to 353 K. Small aliquots of EtOH were periodically added to the solution during the heating process to prolong the reaction time. The resulting mixture was filtered and left for crystallization at room temperature. Suitable crystals for X-ray diffraction measuraments were obtained in the course of two weeks.

### Refinement

C-bound H-atoms were placed in calculated positions and were included in the refinement in the riding model approximation, *U_iso_*(H) = 1.5*U_eq_*(C) for methyl group carbon atoms, *U_iso_*(H) = 1.2*U_eq_*(C) for the other carbon atoms. O-bound H atoms were located in a difference Fourier map and were refined with distance constraints of O—H = 0.84 Å, *U_iso_*(H) = 1.2*U_eq_*(O). The two highest peaks in the difference map are 1.33 and 0.97 e^-^ per Å^3^, respectively. The corresponding distances to the nearest atom, Sm1, are 0.866 and 0.864 Å.

### Crystal data

**Table:**

[Sm(NO_3_)_3_(C_2_H_6_O)(C_4_H_10_O_4_)]	*F*(000) = 988
*M**_r_* = 504.57	*D*_x_ = 2.213 Mg m^−^^3^
Monoclinic, *P*2_1_/*c*	Mo *K*α radiation, λ = 0.71073 Å
Hall symbol: -P 2ybc	Cell parameters from 5882 reflections
*a* = 7.8537 (16) Å	θ = 1.4–27.5°
*b* = 12.875 (3) Å	µ = 3.96 mm^−^^1^
*c* = 15.252 (3) Å	*T* = 173 K
β = 100.92 (3)°	Block, colorless
*V* = 1514.4 (5) Å^3^	0.27 × 0.21 × 0.16 mm
*Z* = 4	

### Data collection

**Table:**

Rigaku Saturn724+ CCD diffractometer	3446 independent reflections
Radiation source: fine-focus sealed tube	3315 reflections with *I* > 2σ(*I*)
Graphite monochromator	*R*_int_ = 0.035
Detector resolution: 28.5714 pixels mm^-1^	θ_max_ = 27.5°, θ_min_ = 2.1°
ω scans fixed at = 45°	*h* = −10→10
Absorption correction: multi-scan (*CrystalClear*; Rigaku, 2007)	*k* = −16→15
*T*_min_ = 0.488, *T*_max_ = 1.000	*l* = −19→19
10349 measured reflections	

### Refinement

**Table:**

Refinement on *F*^2^	Primary atom site location: structure-invariant direct methods
Least-squares matrix: full	Secondary atom site location: difference Fourier map
*R*[*F*^2^ > 2σ(*F*^2^)] = 0.029	Hydrogen site location: inferred from neighbouring sites
*wR*(*F*^2^) = 0.057	H-atom parameters constrained
*S* = 1.22	*w* = 1/[σ^2^(*F*_o_^2^) + (0.010*P*)^2^ + 2.6923*P*] where *P* = (*F*_o_^2^ + 2*F*_c_^2^)/3
3446 reflections	(Δ/σ)_max_ = 0.001
218 parameters	Δρ_max_ = 1.33 e Å^−^^3^
0 restraints	Δρ_min_ = −0.72 e Å^−^^3^

### Special details

**Table:**

Geometry. All esds (except the esd in the dihedral angle between two l.s. planes) are estimated using the full covariance matrix. The cell esds are taken into account individually in the estimation of esds in distances, angles and torsion angles; correlations between esds in cell parameters are only used when they are defined by crystal symmetry. An approximate (isotropic) treatment of cell esds is used for estimating esds involving l.s. planes.
Refinement. Refinement of F^2^ against ALL reflections. The weighted R-factor wR and goodness of fit S are based on F^2^, conventional R-factors R are based on F, with F set to zero for negative F^2^. The threshold expression of F^2^ > 2sigma(F^2^) is used only for calculating R-factors(gt) etc. and is not relevant to the choice of reflections for refinement. R-factors based on F^2^ are statistically about twice as large as those based on F, and R- factors based on ALL data will be even larger.

### Fractional atomic coordinates and isotropic or equivalent isotropic displacement parameters (Å^2^)

**Table:**

	*x*	*y*	*z*	*U*_iso_*/*U*_eq_	
Sm1	0.12473 (2)	0.103866 (13)	0.245568 (11)	0.01311 (6)	
O2	−0.1453 (3)	0.19005 (18)	0.26026 (15)	0.0152 (5)	
H2	−0.1627	0.2536	0.2497	0.018*	
O7	0.1854 (3)	−0.09504 (18)	0.26433 (17)	0.0191 (5)	
O12	0.1760 (3)	0.2978 (2)	0.23049 (18)	0.0226 (6)	
O9	−0.0707 (3)	0.0131 (2)	0.11892 (18)	0.0237 (6)	
O3	−0.0744 (3)	0.00648 (18)	0.32999 (16)	0.0172 (5)	
H3	−0.1087	−0.0539	0.3152	0.021*	
O1	0.1328 (3)	0.17252 (18)	0.39375 (16)	0.0171 (5)	
H1	0.1753	0.1387	0.4398	0.021*	
O10	−0.0309 (3)	0.1772 (2)	0.10178 (17)	0.0219 (5)	
O14	0.4196 (3)	0.3751 (2)	0.2861 (2)	0.0298 (6)	
O11	−0.2048 (3)	0.0930 (2)	−0.00145 (18)	0.0284 (6)	
O6	0.3562 (3)	0.00887 (19)	0.35122 (17)	0.0202 (5)	
O4	−0.2725 (3)	−0.0768 (2)	0.45451 (17)	0.0217 (5)	
H4	−0.3741	−0.0886	0.4274	0.026*	
N1	0.3140 (4)	−0.0835 (2)	0.3280 (2)	0.0187 (6)	
N2	−0.1050 (4)	0.0942 (2)	0.0709 (2)	0.0209 (7)	
O13	0.3997 (3)	0.20661 (19)	0.28786 (18)	0.0233 (6)	
O8	0.3934 (4)	−0.1577 (2)	0.36532 (19)	0.0288 (6)	
O5	0.3062 (3)	0.0520 (2)	0.14084 (17)	0.0225 (6)	
H5	0.2654	0.0050	0.1047	0.027*	
N3	0.3355 (4)	0.2958 (2)	0.2693 (2)	0.0180 (6)	
C5	0.4524 (4)	0.0950 (3)	0.1065 (3)	0.0218 (8)	
H5A	0.5248	0.0378	0.0904	0.026*	
H5B	0.5250	0.1374	0.1535	0.026*	
C3	−0.2160 (4)	0.0631 (3)	0.3569 (2)	0.0155 (7)	
H3A	−0.3252	0.0467	0.3137	0.019*	
C4	−0.2402 (4)	0.0320 (3)	0.4494 (2)	0.0195 (7)	
H4A	−0.1348	0.0504	0.4933	0.023*	
H4B	−0.3388	0.0712	0.4650	0.023*	
C2	−0.1785 (4)	0.1780 (3)	0.3500 (2)	0.0166 (7)	
H2A	−0.2836	0.2190	0.3562	0.020*	
C1	−0.0225 (4)	0.2200 (3)	0.4137 (2)	0.0191 (7)	
H1A	−0.0164	0.2964	0.4072	0.023*	
H1B	−0.0328	0.2042	0.4760	0.023*	
C6	0.3896 (5)	0.1610 (3)	0.0262 (3)	0.0275 (9)	
H6A	0.3223	0.1182	−0.0212	0.041*	
H6B	0.4892	0.1912	0.0052	0.041*	
H6C	0.3162	0.2168	0.0421	0.041*	

### Atomic displacement parameters (Å^2^)

**Table:**

	*U*^11^	*U*^22^	*U*^33^	*U*^12^	*U*^13^	*U*^23^
Sm1	0.01387 (8)	0.01135 (10)	0.01389 (10)	−0.00053 (6)	0.00207 (6)	−0.00031 (7)
O2	0.0215 (12)	0.0087 (12)	0.0153 (12)	0.0035 (9)	0.0037 (9)	0.0039 (10)
O7	0.0174 (11)	0.0155 (13)	0.0231 (14)	−0.0022 (9)	0.0010 (10)	−0.0016 (11)
O12	0.0181 (12)	0.0197 (14)	0.0275 (14)	−0.0009 (10)	−0.0017 (10)	0.0055 (11)
O9	0.0284 (13)	0.0166 (13)	0.0248 (14)	−0.0026 (10)	0.0019 (11)	0.0012 (12)
O3	0.0183 (11)	0.0105 (12)	0.0238 (13)	−0.0014 (9)	0.0068 (9)	−0.0022 (10)
O1	0.0166 (11)	0.0180 (13)	0.0160 (12)	0.0031 (9)	0.0012 (9)	−0.0009 (10)
O10	0.0270 (13)	0.0205 (14)	0.0164 (13)	−0.0053 (10)	−0.0001 (10)	0.0012 (11)
O14	0.0291 (15)	0.0187 (14)	0.0410 (18)	−0.0112 (11)	0.0052 (12)	−0.0063 (13)
O11	0.0265 (14)	0.0382 (17)	0.0175 (14)	−0.0043 (12)	−0.0032 (10)	−0.0024 (13)
O6	0.0203 (12)	0.0134 (13)	0.0251 (14)	−0.0014 (9)	−0.0003 (10)	−0.0033 (11)
O4	0.0221 (12)	0.0227 (14)	0.0191 (13)	−0.0042 (10)	0.0007 (10)	0.0073 (11)
N1	0.0196 (14)	0.0170 (16)	0.0195 (16)	0.0000 (11)	0.0033 (11)	−0.0010 (13)
N2	0.0178 (14)	0.0254 (18)	0.0192 (16)	−0.0023 (12)	0.0026 (11)	−0.0017 (14)
O13	0.0179 (12)	0.0166 (13)	0.0341 (15)	−0.0003 (10)	0.0016 (10)	0.0005 (12)
O8	0.0349 (15)	0.0151 (14)	0.0336 (16)	0.0075 (11)	−0.0005 (12)	0.0065 (12)
O5	0.0235 (13)	0.0224 (14)	0.0238 (14)	−0.0037 (10)	0.0102 (10)	−0.0061 (12)
N3	0.0186 (14)	0.0140 (15)	0.0216 (16)	−0.0031 (11)	0.0045 (11)	0.0005 (13)
C5	0.0175 (16)	0.024 (2)	0.026 (2)	0.0004 (14)	0.0094 (14)	0.0040 (17)
C3	0.0139 (15)	0.0161 (18)	0.0167 (17)	0.0029 (12)	0.0033 (12)	0.0012 (14)
C4	0.0222 (17)	0.0183 (19)	0.0178 (18)	−0.0003 (14)	0.0036 (13)	0.0040 (15)
C2	0.0175 (15)	0.0160 (18)	0.0176 (18)	0.0031 (13)	0.0066 (13)	0.0007 (15)
C1	0.0207 (17)	0.0159 (18)	0.0215 (19)	0.0023 (13)	0.0062 (14)	−0.0031 (15)
C6	0.035 (2)	0.023 (2)	0.023 (2)	−0.0038 (16)	0.0037 (16)	0.0020 (17)

### Geometric parameters (Å, º)

**Table:**

Sm1—O1	2.416 (2)	O6—N1	1.267 (4)
Sm1—O5	2.427 (3)	O4—C4	1.429 (4)
Sm1—O2	2.441 (2)	O4—H4	0.8400
Sm1—O10	2.486 (3)	N1—O8	1.221 (4)
Sm1—O6	2.507 (2)	O13—N3	1.265 (4)
Sm1—O13	2.511 (2)	O5—C5	1.459 (4)
Sm1—O9	2.516 (3)	O5—H5	0.8400
Sm1—O3	2.537 (2)	C5—C6	1.496 (5)
Sm1—O12	2.547 (3)	C5—H5A	0.9900
Sm1—O7	2.611 (2)	C5—H5B	0.9900
O2—C2	1.448 (4)	C3—C4	1.512 (5)
O2—H2	0.8400	C3—C2	1.516 (5)
O7—N1	1.270 (4)	C3—H3A	1.0000
O12—N3	1.280 (4)	C4—H4A	0.9900
O9—N2	1.275 (4)	C4—H4B	0.9900
O3—C3	1.453 (4)	C2—C1	1.512 (5)
O3—H3	0.8400	C2—H2A	1.0000
O1—C1	1.448 (4)	C1—H1A	0.9900
O1—H1	0.8400	C1—H1B	0.9900
O10—N2	1.265 (4)	C6—H6A	0.9800
O14—N3	1.216 (4)	C6—H6B	0.9800
O11—N2	1.227 (4)	C6—H6C	0.9800
			
O1—Sm1—O5	143.28 (8)	C1—O1—H1	105.1
O1—Sm1—O2	67.50 (8)	Sm1—O1—H1	121.9
O5—Sm1—O2	144.50 (8)	N2—O10—Sm1	97.0 (2)
O1—Sm1—O10	127.41 (8)	N1—O6—Sm1	99.15 (18)
O5—Sm1—O10	77.06 (9)	C4—O4—H4	108.2
O2—Sm1—O10	67.52 (8)	O8—N1—O6	121.3 (3)
O1—Sm1—O6	71.93 (8)	O8—N1—O7	121.8 (3)
O5—Sm1—O6	81.04 (9)	O6—N1—O7	116.8 (3)
O2—Sm1—O6	134.38 (8)	O11—N2—O10	121.1 (3)
O10—Sm1—O6	158.09 (9)	O11—N2—O9	122.4 (3)
O1—Sm1—O13	72.42 (9)	O10—N2—O9	116.5 (3)
O5—Sm1—O13	74.36 (9)	N3—O13—Sm1	97.69 (18)
O2—Sm1—O13	117.19 (8)	C5—O5—Sm1	137.0 (2)
O10—Sm1—O13	106.37 (9)	C5—O5—H5	105.5
O6—Sm1—O13	66.94 (8)	Sm1—O5—H5	115.6
O1—Sm1—O9	142.39 (8)	O14—N3—O13	122.5 (3)
O5—Sm1—O9	73.49 (9)	O14—N3—O12	121.7 (3)
O2—Sm1—O9	82.37 (8)	O13—N3—O12	115.8 (3)
O10—Sm1—O9	51.15 (8)	O5—C5—C6	110.4 (3)
O6—Sm1—O9	121.97 (8)	O5—C5—H5A	109.6
O13—Sm1—O9	144.30 (9)	C6—C5—H5A	109.6
O1—Sm1—O3	67.40 (8)	O5—C5—H5B	109.6
O5—Sm1—O3	133.87 (8)	C6—C5—H5B	109.6
O2—Sm1—O3	63.15 (8)	H5A—C5—H5B	108.1
O10—Sm1—O3	112.83 (8)	O3—C3—C4	111.6 (3)
O6—Sm1—O3	82.77 (8)	O3—C3—C2	107.5 (3)
O13—Sm1—O3	135.47 (8)	C4—C3—C2	112.6 (3)
O9—Sm1—O3	79.34 (8)	O3—C3—H3A	108.3
O1—Sm1—O12	75.47 (8)	C4—C3—H3A	108.3
O5—Sm1—O12	95.05 (9)	C2—C3—H3A	108.3
O2—Sm1—O12	73.59 (8)	O4—C4—C3	111.5 (3)
O10—Sm1—O12	66.89 (8)	O4—C4—H4A	109.3
O6—Sm1—O12	115.38 (8)	C3—C4—H4A	109.3
O13—Sm1—O12	50.47 (8)	O4—C4—H4B	109.3
O9—Sm1—O12	118.03 (8)	C3—C4—H4B	109.3
O3—Sm1—O12	130.81 (8)	H4A—C4—H4B	108.0
O1—Sm1—O7	106.52 (8)	O2—C2—C1	107.5 (3)
O5—Sm1—O7	71.60 (8)	O2—C2—C3	104.0 (3)
O2—Sm1—O7	125.41 (8)	C1—C2—C3	116.7 (3)
O10—Sm1—O7	121.23 (8)	O2—C2—H2A	109.5
O6—Sm1—O7	49.91 (8)	C1—C2—H2A	109.5
O13—Sm1—O7	111.01 (8)	C3—C2—H2A	109.5
O9—Sm1—O7	72.58 (8)	O1—C1—C2	109.1 (3)
O3—Sm1—O7	64.96 (8)	O1—C1—H1A	109.9
O12—Sm1—O7	160.58 (8)	C2—C1—H1A	109.9
C2—O2—Sm1	110.65 (18)	O1—C1—H1B	109.9
C2—O2—H2	103.5	C2—C1—H1B	109.9
Sm1—O2—H2	122.5	H1A—C1—H1B	108.3
N1—O7—Sm1	94.10 (18)	C5—C6—H6A	109.5
N3—O12—Sm1	95.50 (19)	C5—C6—H6B	109.5
N2—O9—Sm1	95.30 (19)	H6A—C6—H6B	109.5
C3—O3—Sm1	118.28 (18)	C5—C6—H6C	109.5
C3—O3—H3	108.5	H6A—C6—H6C	109.5
Sm1—O3—H3	121.2	H6B—C6—H6C	109.5
C1—O1—Sm1	118.48 (19)		

### Hydrogen-bond geometry (Å, º)

**Table:**

*D*—H···*A*	*D*—H	H···*A*	*D*···*A*	*D*—H···*A*
O1—H1···O4^i^	0.84	1.83	2.668 (3)	175
O2—H2···O7^ii^	0.84	1.96	2.802 (3)	174
O2—H2···O8^ii^	0.84	2.54	3.146 (4)	130
O3—H3···O12^iii^	0.84	2.07	2.903 (3)	174
O4—H4···O8^iv^	0.84	2.09	2.910 (4)	165
O4—H4···O6^iv^	0.84	2.55	3.235 (3)	140
O5—H5···O11^v^	0.84	2.00	2.827 (4)	167

Symmetry codes: (i) −*x*, −*y*, −*z*+1; (ii) −*x*, *y*+1/2, −*z*+1/2; (iii) −*x*, *y*−1/2, −*z*+1/2; (iv) *x*−1, *y*, *z*; (v) −*x*, −*y*, −*z*.
